# External Corrosion Behavior of Steel/GFRP Composite Pipes in Harsh Conditions

**DOI:** 10.3390/ma14216501

**Published:** 2021-10-29

**Authors:** Fatima Ghassan Alabtah, Elsadig Mahdi, Marwan Khraisheh

**Affiliations:** 1 Mechanical Engineering Program, Texas A&M University at Qatar, Doha P.O. Box 23874, Qatar; marwan.khraisheh@qatar.tamu.edu; 2 Department of Mechanical and Industrial Engineering, Qatar University, Doha P.O. Box 2713, Qatar; elsadigms@qu.edu.qa

**Keywords:** corrosion, environmental degradation, glass fiber–epoxy composites, acids attack, hybrid pipes

## Abstract

In this study, we report on the corrosion behavior of hybrid steel/glass fiber-reinforced polymer (GFRP) composite pipes under harsh corrosive conditions for prolonged durations. Specimens were immersed in highly concentrated solutions of hydrochloric acid, sodium chloride, and sulfuric acid for durations up to one year. Detailed qualitative analysis using scanning electron microscopy (SEM), X-ray diffraction analysis (XRD), and energy-dispersive X-ray spectroscopy (EDX) is presented. It is shown that the hybrid pipes have excellent corrosion resistance with a corrosion rate of less than 1% of the corrosion rate for conventional steel pipes. That low corrosion rate can be attributed to the formation of pores in the GFRP layer due to increased absorption and saturation moisture in the material with increased soaking time. This can be reduced or even prevented through a more controlled process for fabricating the protective layers. These promising results call for more utilization of GFRP protective layers in novel design concepts to control corrosion.

## 1. Introduction

Corrosion is a harmful attack on metallic structures and pipes that leads to catastrophic failures resulting in significant economic and environmental losses. Industries at high risk of corrosion include oil and gas, power plants, water treatment plants, and chemical process facilities. In the oil and gas industry, external corrosion results from salt and seawater, while chemical solvents cause internal corrosion [1]. Significant efforts and financial resources are devoted to addressing corrosion by developing new methods, materials, and technologies that can eliminate or delay corrosion of metallic components [2]. Both corrosion and abrasion cause significant losses and decrease the structural integrity of pipelines [3]. For corrosion to happen, three critical components must exist. The first component is the anode that represents the metal under corrosion. The second is the electrolyte, which is the corrosive medium that transmits the electrons from the anodic site to the cathodic site. The third is the cathode, which is the electrical conductor [4]. Oil and gas generally transmit several impurities that are corrosive under many conditions. These impurities might contain carbon dioxide (CO_2_) and hydrogen sulfide (H_2_S) [5]. In addition, the effect of Chloride ion (Cl^−^) concentration on corrosion rates and the interaction between Cl^−^ and CO_2_ towards carbon steel corrosion, where chloride ion penetrates the imperfection and begins the process of pit initiation [6,7,8,9].

Acidic attacks on metal pipes can come from the soil in which the pipe is buried, which will cause corrosion damage for the surfaces of oil and gas pipelines. The degradation of metals is followed regularly by the retrogradation of mechanical properties such as strength and ductility. This may lead to the weakening of material and ultimate failure [10,11]. External corrosion is generally slowed down using organic coating and cathodic protection [12,13]. Various polymeric coating types were used to protect pipeline surfaces against corrosion. The most common polymeric coatings are epoxy resins that are widely used because of their excellent adhesion properties [14]. Hsissou et al. developed different novel epoxy polymers. They investigated their anticorrosive performance for carbon steel in different corrosive media. The results indicated that the polymeric coatings employed have high protective efficiency and significantly reduced the corrosion rate of carbon steel [15,16]. Inner corrosion is one of the critical factors affecting the integrity of oil and gas pipelines. Non-destructive analysis such as ultrasound and magnetic current is used to detect and measure the inner corrosion damage [17]. Corrosion inhibitors are widely used to fight internal corrosion [18]. Tan et al. and Zuo et al. investigated the inhibitive effect of different eco-friendly and mixed-type inhibitors for metals in corrosive media. The results revealed excellent corrosion inhibition performance maintained in a wide temperature range [19,20,21,22,23,24].

Although effective for the short term, these practices suffer from sustained efficiency in the long term and from added cost to repeated maintenance and repair. Alternatively, composite pipe materials have been introduced and widely accepted in the oil and gas industry [25]. Fiber-reinforced polymer (FRP) composites are frequently utilized in the chemical industry for pipelines and storage tanks [26,27,28]. Although they offer protection against corrosion, they are limited to low to moderate pressure applications [29] and suffer from cracking and mechanical failures. Experts in the oil and gas industry agree that future transmission pipelines will have to operate at higher pressures and decrease the cost of corrosion. To meet the increased demands, maintain safety and reliability, and be competitive, pipeline designers and operators are looking for alternative pipe materials to conventional metallic and pure composite pipelines. Most of the reported studies focused on the corrosion behavior of coated carbon steel with polymers and corrosion inhibition materials [30,31,32,33]. More recently, pipes made of steel with external warps of glass or carbon-based composite layers have been introduced [29]. The idea behind the external layers of composite materials is to prevent/eliminate external corrosion and add extra mechanical strength to the pipe [34]. GFR/epoxy are the most used materials for overwrapping metallic pipes due to their low cost, good mechanical and insulating properties [35,36], and deterioration resistance, especially when they interact with sweet or salty water. Numerous studies experimentally tested the use of glass-reinforced polymer (GRP) pipes in different applications [25]. Glass fiber-reinforced epoxy composite pipes are used in submarine applications, natural gas and oil transportation lines, and the transfer of chemical liquids, especially in the transport of pressurized fluids, where changes in the strength of the pipes are essential [37]. In terms of costs, the approximate prices for GFR/epoxy range from USD 1.9 to 3.9 per kilogram. The principal advantages of GFR/epoxy materials are the relationship between their low cost, high tensile strength, high chemical resistance, and insulating properties [38].

Several pieces of research have focused on the design process and testing of durability for GFRP pipeline in complex marine environments [39,40], where GFRP has been widely accepted for seawater applications. Lea et al. [41] mentioned that using GFRP pipes for seawater applications after several years indicated no material loss in the pipes. Zhou et al. [42] investigated the effect of using CFRP and GFRP composites on corrosion resistance of innovative steel–FRP composite bars in NaCl solution, where it was found that the corrosion rates of carbon type and glass type were less than 1/10 and 1/100, respectively, than that of an ordinary steel bar. However, when exposed to acidic corrosive media or harsh oil-well conditions, the GFRP may be degraded due to abrasion, change in brittleness, delamination, or separation of fiber from the matrix, and degradation of the matrix because of the highly corrosive environments [25]. Some researchers investigated the harsh environmental effect on the GFR/epoxy mechanical properties, durability, and performance [43]. Li et al. [44] investigated the mechanical properties and service life prediction of carbon/glass hybrid rods exposed to harsh oil-well conditions. It was speculated that a severe structural degradation of the hybrid rod might occur after long-term exposure, where increased resin voids and fiber/resin interface debonding were documented, especially for carbon fibers. The authors revealed that this is mainly attributed to the hydrolysis and plasticization of resin. Both Amaro et al. [45] and Kajorncheappunngam et al. [46] studied the effects of hydrochloric acid (HCl) on glass/epoxy composites, and results revealed that the flexural strength and the flexural modulus decrease with the exposure time. Kotnarowska [47] studied the destruction of epoxy due to sulfuric acid solution and proved the generation of pores in the aged coatings.

Despite these studies, the long-term performance of the GFRP as a strengthening and protection layer for steel pipes under a wide range of corrosive environments still requires a comprehensive investigation. In the present study, we evaluate the corrosion behavior of GFRP/steel under long-term- up to one year- immersion tests in different acidic environments, including hydrochloric acid, sodium chloride, and sulfuric acid solutions with 0.5M concentrations. Our goal is to design a novel hybrid piping system using GFRP protection layers to prevent both external and internal corrosion. The experiments were designed to expose the outer protective layer to acidic environments without exposing the interior of the pipes.

## 2. Materials and Methods

### 2.1. Materials

The base material was ASTM A53 carbon steel alloy. The base material’s chemical composition and mechanical properties are presented in Table 1 and Table 2, respectively. Carbon steel pipes were overwrapped with a composite material that consisted of glass fiber and epoxy resin. E-glass fiber is commonly used because of its low cost [48]. Table 3 presents the mechanical properties of the used glass fibers. The polymeric matrix is composed of epoxy resin (EL2) and hardener (AT30) that are clear and have high purity. The matrix comprises 100 parts mass of the epoxy resin (EL2) and 30 parts mass of curing agent (AT30). Matrix resin with low diffusivity can theoretically protect the fibers from direct contact with the environmental liquid over a long time span [49].

### 2.2. Specimen Preparation

**Figure 1:**
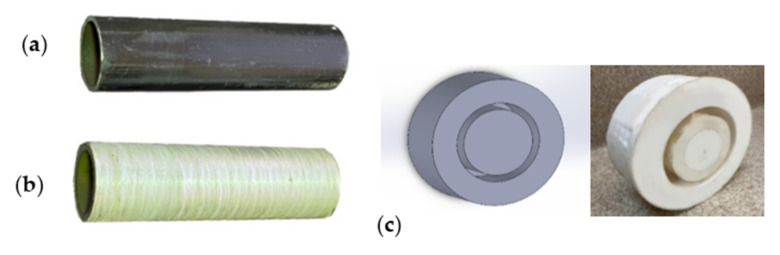
The prepared specimens, (**a**) steel pipe, (**b**) GFRP overwrapped steel pipe, and (**c**) Teflon cups, to close the two ends of the pipes.

The carbon steel pipes had a 6 cm outer diameter, a thickness of 3 mm, and 20 cm for each tested specimen (Figure 1a). The five-axis filament-winding machine was used to overwrap the steel pipes with the glass FRP material. The filament winding machine is the most utilized fabrication method in overwrapping steel pipes [50]. The fibers’ orientation angle in all the samples was unidirectional at 90° (Figure 1b). The average thickness of the GFRP layer in all the specimens was 2.5 mm ± 0.1 mm, consisting of eight layers. The pipe specimens were closed and sealed using Teflon cups (Figure 1c) and chemical-resistant sealing to prevent leakage to the inner steel layer and ensure that the contact is only between the GFRP composite and the corrosive solution. The cups were designed on SOLIDWORKS (SolidWorks, Concord, MA, USA) and fabricated on the CNC machine (DMG Mori, Koto, Tokyo, Japan).

### 2.3. Immersion Corrosion Test

**Figure 2:**
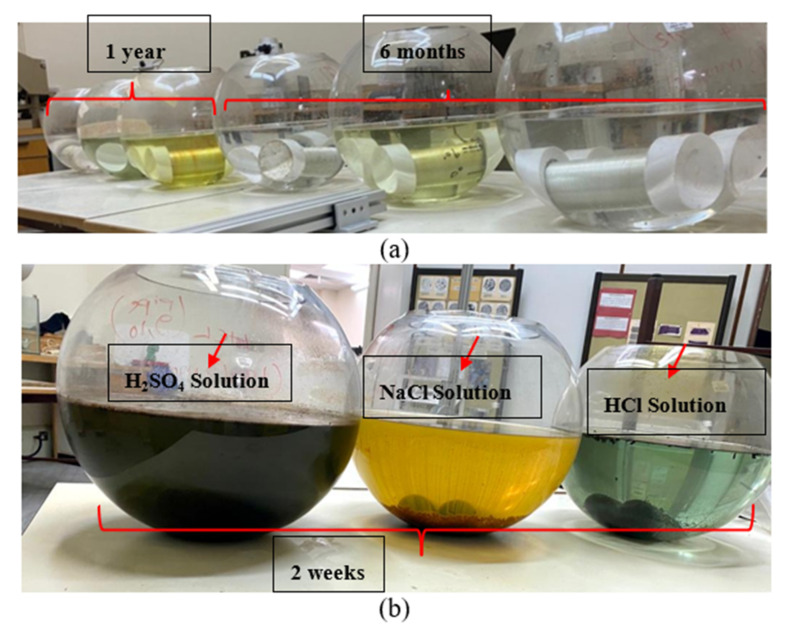
(**a**) GFRP/steel pipes after one year and six months, (**b**) steel pipes after two weeks of immersion in acidic environments.

To evaluate the performance of hybrid composite/metallic pipes and their corrosion properties under harsh corrosive conditions, GFRP/steel specimens were prepared and immersed in different corrosive environments. GFRP/steel pipes’ corrosion aspects were evaluated in hydrochloric acid 37%, sodium chloride, and sulfuric acid 95% solutions with 0.5 M concentration. The specimens were closed and sealed using Teflon cups and chemical resistant sealing to prevent leakage to the inner steel layer and ensure that the contact is only between the GFRP composite and the corrosive solution. The GFRP/steel pipes were immersed in a glass container containing the corrosive solutions and monitored for six months and one year (Figure 2a). Simultaneously, the steel pipes without any coating were immersed for two weeks (Figure 2b).

According to ASTM TM0169/G31 [51], duplicate test specimens were exposed in each test, and evaporation losses were controlled by the frequent addition of appropriate solutions to maintain the original volume within ±1%.

## 3. Results and Discussion

**Figure 3:**
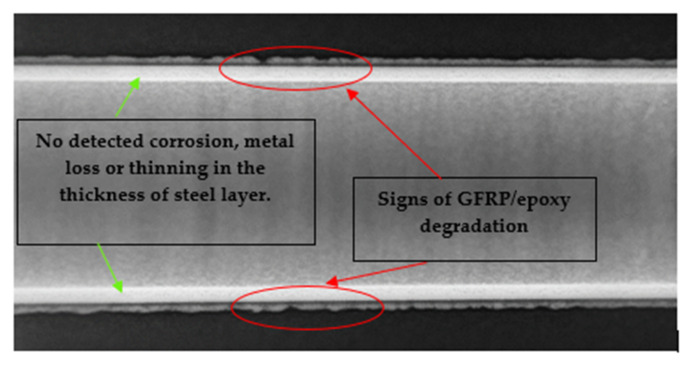
Gamma-ray radiography for the GFRP/steel pipe immersed in HCl solution for 1 year.

Table 4 shows the photos for immersion test containers for all the specimens. Some yellowing signs have been observed at the surface of the GFRP/steel pipes immersed in the HCl solution. Gamma-ray radiography was used to investigate the FRP layer in this specimen and check any sign of corrosion in the steel layer before peeling off the GFRP layer (Figure 3) (diffusion channel). The radiography images revealed no detected corrosion, metal loss, or thinning in the thickness of the steel layer. However, some degradation for the FRP layer and dissolving of epoxy was observed due to the long-term interaction with the highly concentrated hydrochloric acid solution. According to Krauklis et al. [52], the cause of yellowing in the epoxy layer could be related to the irreversible aging mechanism. When the color of the epoxy surface becomes yellow, there is oxidation in the epoxy resin chains [53].

After taking all the immersed pipe out of the solution, the Teflon cups were removed, and the FRP layer was cut and peeled off from the steel pipes. The corrosion condition was observed and qualitatively analyzed by visual inspection, scanning electron microscopy (SEM), X-ray diffraction analysis (XRD), and energy-dispersive X-ray spectroscopy (EDX). Photos for the immersed pipes after the end of the immersing test are presented in Table 5. By visual inspection, no areas of pitting corrosion were noticed in all the overwrapped steel pipes.

No significant weight loss was observed in the steel pipes that were overwrapped with the GFRP layer. Simultaneously, the steel pipes immersed without any coating had a significant weight loss after 2 weeks only. The mass loss during the test period can be used as the principal measure of corrosion [54]. Before weighing the corroded pipes, they were cleaned according to the G1-03 ASTM standard [55], which mentions that the ideal cleaning procedure is when cleaning removes only corrosion products and does not remove any base metal. A soft metallic bristle brush was used in the cleaning of the corrosion products. After the corroded test specimens had been cleaned, their masses were measured with an accuracy corresponding to the original mass measurements.

The steel pipes that were immersed in the corrosive environments witnessed high weight loss in a brief period. The pipes lost 20%, 10%, and 1% of their total weight when immersed in 0.5 M sulfuric acid, hydrochloric acid, and sodium chloride solutions, respectively, in 2 weeks. One can calculate the average corrosion rate by the following equation [55].

Corrosion rate = (K × W)/(A × T × D)(1)

where: K = a constant = 8.76 × $10^{4}$, T = time of exposure in hours, A = area of exposure = (outer surface area of the pipe + inner surface area of the pipe + 2 × base area) (for non-coated steel pipe), and only outer surface area for GFRP/steel pipes. W = mass loss in g, and D = density in g/cm^3^ = 7.85 g/${\mathrm{cm}}^{3}$. Table 6 presents the corrosion rates for the immersed steel pipes in different solutions.

According to the qualitative categorization of carbon steel corrosion rates for oil production systems, any corrosion rate above 0.38 mm/y is considered severe pitting corrosion, a catastrophic localized steel failure that produces severe global economic losses [56]. Moreover, any corrosion rate below 0.025 mm/y is considered low general corrosion [30], where general corrosion or rusting is considered a uniform corrosion process, in which micro corrosion cells are activated at the corroded area. For the GFRP/steel pipes, no weight loss was detected in the case of NaCl solution, which means that the GFRP layer eliminated any possible corrosion in the base metal. Additionally, for HCl and H_2_SO_4_, the corrosion rate is considered very low compared to the corrosion rate of the non-coated steel (less than 1/100 of the conventional steel pipes corrosion rate).

### 3.1. SEM and EDX

**Figure 4:**
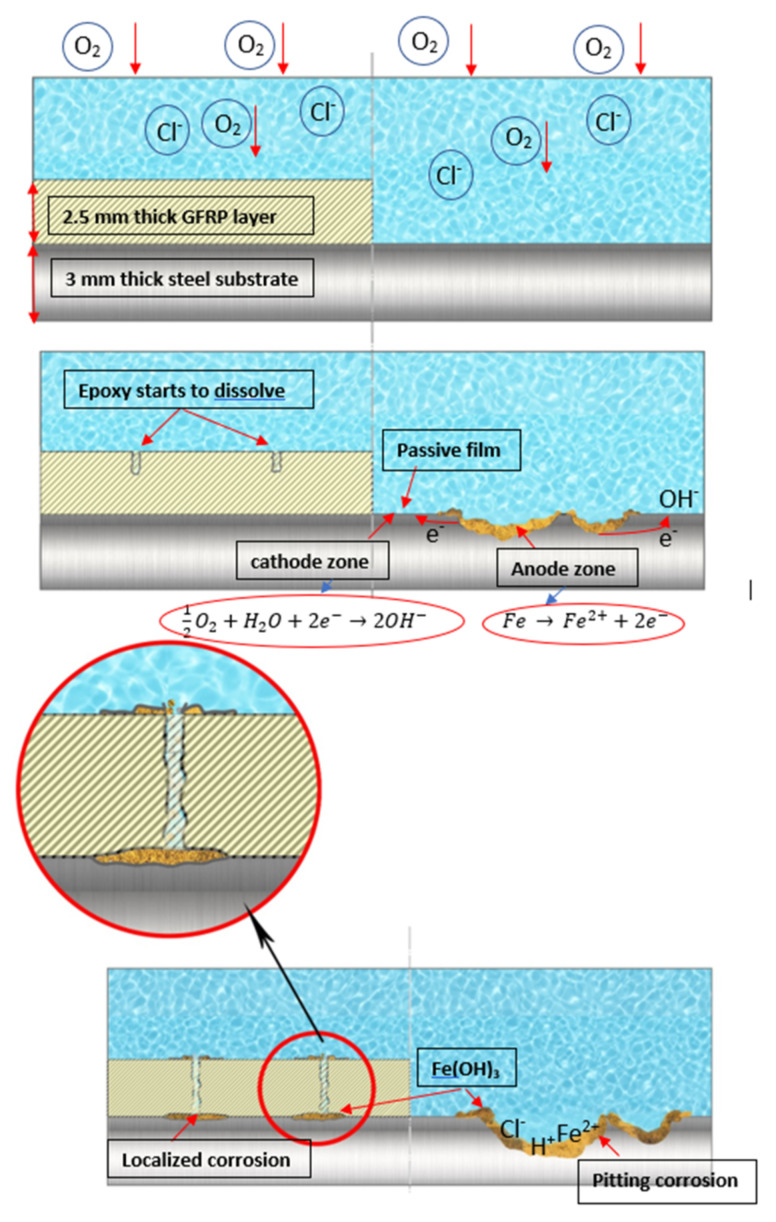
Schematic for the development of corrosion on uncoated steel vs. GFRP/steel in corrosive solution.

Table 7 presents the surface analysis (SEM/EDX) that shows the participation of the different alloying elements in each tested specimen according to the alloy constituents for the immersed steel pipes before and after immersion in the different solutions at a magnification of 5000×. The first row shows the SEM images for the steel after polishing, where the steel surface is very flat, and the traces are shown results from sanding by sandpaper. The second and third rows present the SEM images for the pipes that were overwrapped by GFRP and immersed in different solutions for six months and one year, respectively. It could be observed that the surface of the steel that was immersed in sodium chloride solutions is smooth and clean, and there is no evidence of any corrosion reaction, which proves the functionality of the GFRP layer, while for the cases of HCl and H_2_SO_4_ immersion, it could be noticed that the surface is rough due to the removal effect of the GFR/epoxy layer and the presence of some remnant epoxy particles. The fourth row shows the cases of non-coated immersed steel pipes. The universal corrosion products, some of which develop and blossom to be corrosion flowers, covered the exposed specimens totally, as shown in the SEM images of the immersed steel in HCl and H_2_SO_4_. Moreover, the porous lamellar structure is detected in all the immersed specimens without coating, which results from the pitting corrosion feature, especially when immersed in HCl solution. The critical factor leading to stress cracking corrosion and pitting corrosion of pipeline steel is chloride ion reactions. The adsorbed chloride ions on the metal surface will break down the steel’s passivity and consequently upturn the corrosion rate [57]. Figure 4 presents a schematic diagram that demonstrates the pitting corrosion mechanism of pipeline steel in the corrosive environment containing chloride ions, where the negative ions of chloride attract the hydrogen protons to the pitting areas, causing hydrogen protons to accumulate, driving a more significant cathodic reaction outside the pitting and accelerating anodic reaction inside the pitting.

### 3.2. X-ray Diffraction Analysis (XRD)

**Figure 5:**
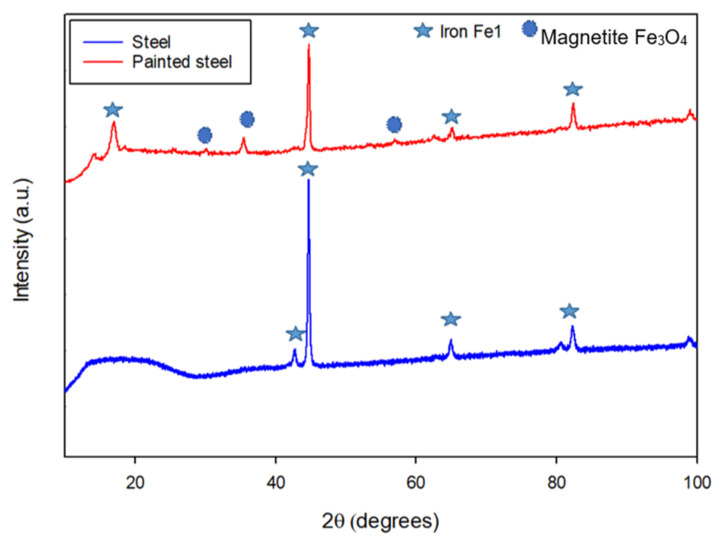
XRD pattern for the steel surface before immersion.

**Figure 6:**
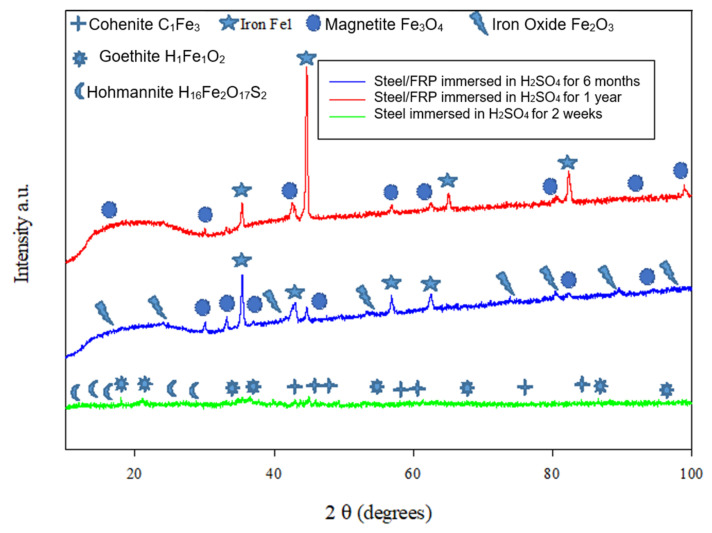
XRD patterns for the steel surfaces after immersion in H_2_SO_4_ solution.

**Figure 7:**
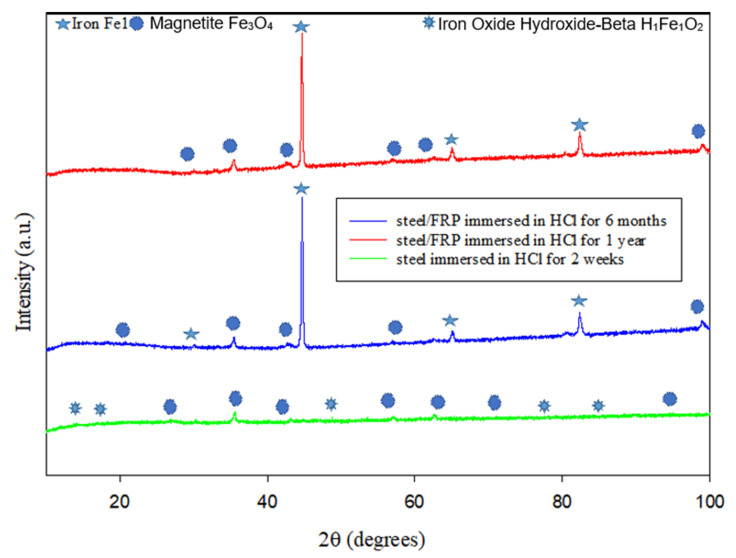
XRD patterns for the steel surfaces after immersion in HCl solution.

**Figure 8:**
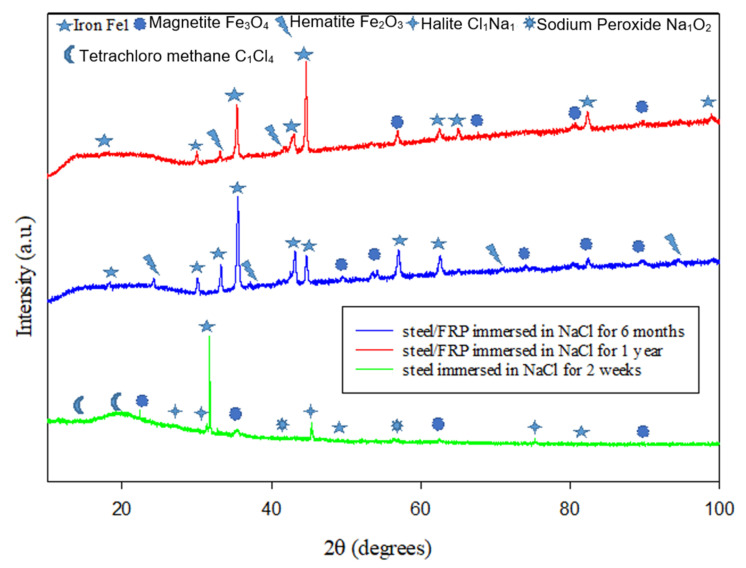
XRD patterns for the steel surfaces after immersion in NaCl solution.

Standard methods for identifications and characterizations of iron oxides have traditionally used X-ray diffraction (XRD). X-ray diffraction analysis was applied to all immersed specimens to detect and characterize any corrosion products’ formation on the surface. Figure 5, Figure 6, Figure 7 and Figure 8 show the XRD patterns representing the signal intensity against 2θ collected from the carbon steel surfaces at the end of each immersion experiment for the three corrosive solutions. Figure 5 patterns reveal that the only crystalline phase detected on the polished steel surface was Iron (Fe) and some low peaks of iron oxide phase as magnetite compound (Fe_3_O_4_) for the painted steel specimen, and no other phases were detected. The most intense peak for both patterns was observed at 2θ=44.79°, and the presence of iron oxide compounds results from the typical oxidation in nature, where the painted pipes were tested as received from the industry without any polishing.

Figure 6 patterns for the steel pipes were overwrapped with a GFRP layer and immersed in sulfuric acid, hydrochloric acid, and sodium chloride solutions for six months and one year. The patterns reveal that the crystalline phases detected are mainly iron Fe1, where the most intense peak in almost all the specimens was observed at 2θ=44.79°, the same as in the new steel specimen. It is also observed that iron oxide compounds were detected in small peaks as magnetite and hematite in all the patterns. Some researchers studied the transformation of iron oxides. They found that it begins with nucleation and growth of goethite (FeOOH), followed by dehydration to hematite (Fe_2_O_3_), and then reduction to magnetite (Fe_3_O_4_) [58]. Simultaneously, the XRD analysis for the uncoated steel that was immersed for two weeks in sulfuric acid solutions exposes that the corrosion products consist mainly of cohenite, goethite, and hohmannite, all of which are compounds that are usually found in iron corrosion scales. Goethite (FeOOH) is an iron oxyhydroxide, and it is the main component of rust [59]; those compounds are predominately present in iron corrosion scales of corroded iron pipe in water distribution systems [60].

Figure 7 reveals that the uncoated steel’s corrosion products immersed in HCl solution consist of magnetite and goethite for two weeks. Iron corrosion is an anoxygenic atmosphere that usually proceeds via goethite (FeOOH) through hematite and magnetite [61]. Magnetite is a familiar kind of iron oxide that forms at room temperature in crevices at steel alloy, where the oxidation product of Fe_3_O_4_ is either γ-Fe_2_O_3_ or α-Fe_2_O_3_, depending on the oxidation temperature [62]. Meanwhile, Figure 8 reveals that the crystalline phases detected for the uncoated steel that was immersed for two weeks in NaCl solution are mainly iron Fe1 with the presence of compounds that consists of halite (Cl_1_Na_1_), sodium peroxide (Na_1_O_2_), and tetrachloromethane (C_1_Cl_4_). Halite is a form of isometric crystals known as rock salt [63] accumulated on the surface of the steel pipe when immersed in sodium chloride solution. Simultaneously, sodium peroxide is produced as the reaction of sodium with oxygen that crystallizes with hexagonal symmetry [64].

## 4. Conclusions

The integrity of GFRP/steel pipes exposed to different highly corrosive environments for prolonged durations up to one year was investigated. Detailed analysis using SEM, XRD, and EDX is presented. The results demonstrated that the GFRP/steel pipes have excellent corrosion resistance compared to uncoated carbon steel pipes. The corrosion rate was estimated to be less than 1% of the corrosion rate for conventional steel pipes. The results are promising, and they indicate that when the protective layer is fabricated with the appropriate resin system, the FRP overwrapped layers will offer excellent resistance to corrosion for a long time and will improve the durability of the pipelines with severe corrosive environments. However, more investigations are needed to determine the best resin system for a particular working fluid. The following conclusions can be drawn:

- The radiography images revealed no detected corrosion, metal loss, or thinning in the thickness of the steel layer.
- For GFRP/steel pipes immersed in NaCl solution, no weight loss was detected after one year immersion, indicating that the GFRP layer eliminated any possible corrosion in the base metal.
- For HCl and H_2_SO_4_ solutions, the corrosion rate was found to be 0.017 and 0.0239 mm/year, respectively, which is considered very low compared to the corrosion rate of the non-coated steel, which was measured to be 4.69 and 8.99 mm/y, respectively.
- The small corrosion rate observed for samples immersed in hydrochloric and sulfuric acid solutions can be explained by the attack of the highly concentrated acidic solutions on the GFRP layer surface, leading to an increased saturation uptake of water in the material. Hence, the absorption and saturation moisture content rates increased with the increase of soaking time, eventually leading to pore formation in the GFRP layer.
- SEM/EDX surface analysis showed the presence of the different alloying elements in each tested specimen. Additionally, it was observed that the surface of the overwrapped steel is generally smooth and clean, which proves the functionality of the GFRP layer. At the same time, the presence of some rough areas is due to the removal effect of the GFR/epoxy layer and the presence of some remnant epoxy particles.
- The porous lamellar structure was detected in all the immersed uncoated steel specimens, resulting from the pitting corrosion feature.
- X-ray diffraction analysis was applied on all immersed specimens, and the corrosion products’ formation on the surface was detected and characterized. The patterns revealed that the crystalline phases detected in the coated steel specimens are mainly iron Fe1. It was also found that the corrosion products for the exposed steel specimens consist mainly of magnetite, cohenite, goethite, and hohmannite.

## Figures and Tables

**Table 1:** Chemical composition (wt.%) of ASTM A53 carbon steel alloy.

Iron	Carbon	Manganese	Phosphorus	Sulfur	Copper	Nickel	Chromium	Molybdenum	Vanadium
96.9	0.3	1.2	0.05	0.045	0.4	0.4	0.4	0.15	0.08

**Table 2:** Mechanical properties of ASTM A53 carbon steel alloy.

Yield Strength (MPa)	Tensile Strength (MPa)	Elongation in 50 mm Min. (%)	Hardness (HRB)
240	415	21	241

**Table 3:** Mechanical properties of glass fiber.

Elastic Modulus (GPa)	Tensile Strength (MPa)	Elongation at Failure (%)	Density (g/cm^3^)
73	3400	4.8	2.56

**Table 4:** Photos for immersion test containers for all the specimens.

Specimen	NaCl Solution	H_2_SO_4_ Solution	HCl Solution
Steel/GFRP pipes after six months	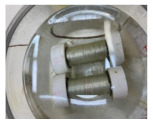	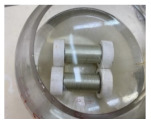	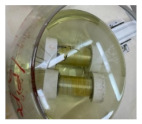
Steel/GFRP pipes after one year	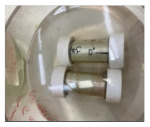	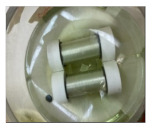	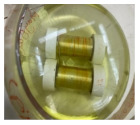
Steel pipes at day 1	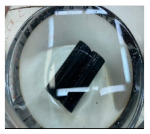	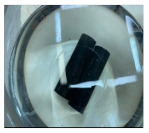	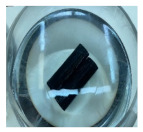
Steel pipes after two weeks	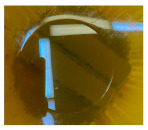	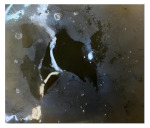	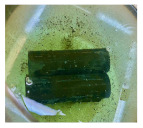

**Table 5:** Photos for the immersed pipes after the end of the immersing test.

Specimen	H_2_SO_4_ Solution	NaCl Solution	HCl Solution
GFRP/steel pipes immersed for six months after removing the GFRP layer	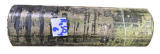	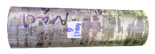	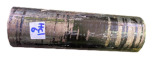
GFRP/steel pipes immersed for one year after removing the GFRP layer	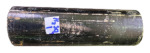	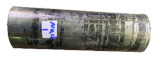	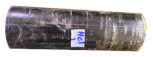
Steel pipes immersed for two weeks	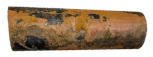	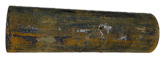	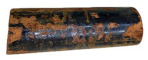

**Table 6:** Corrosion rates for the immersed steel pipes in different solutions.

Specimen	Exposure Time (Hours)	Area of Exposure cm^2^	Mass Loss (g)	Corrosion Rate (mm/y)
Non-coated steel in 0.5 M H_2_SO_4_ solution (2 weeks)	336	742.5	201	8.99
Non-coated steel in 0.5 M HCl solution (2 weeks)	336	742.5	105	4.69
Non-coated steel in 0.5 M NaCl solution (2 weeks)	336	742.5	11	0.49
GFRP/steel in 0.5 M H_2_SO_4_ solution (6 months)	4320	377	5	0.034
GFRP/steel in 0.5 M HCl solution (6 months)	4320	377	3	0.020
GFRP/steel in 0.5 M NaCl solution (6 months)	4320	377	0	0
GFRP/steel in 0.5 M H_2_SO_4_ solution (1 year)	8640	377	7	0.0239
GFRP/steel in 0.5 M HCl solution (1 year)	8640	377	5	0.017
GFRP/steel in 0.5 M NaCl solution (1 year)	8640	377	0	0

**Table 7:** SEM images and EDX analysis for the immersed specimens before and after immersion (5000× magnification).

Specimen	SEM	EDX
New steel pipe (before immersion)	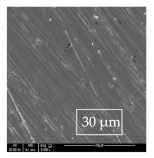	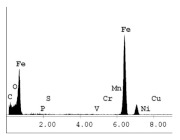
GFRP/steel pipes immersed for six months in NaCl solution after removing the GFRP layer	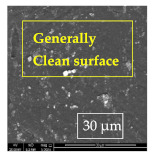	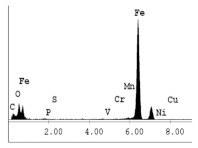
GFRP/steel pipes immersed for six months in H_2_SO_4_ solution after removing the GFRP layer	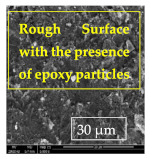	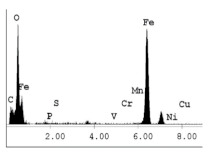
GFRP/steel pipes immersed for six months in HCl solution after removing the GFRP layer	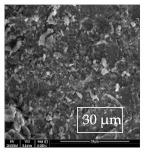	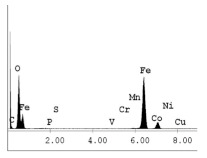
GFRP/steel pipes immersed for one year in NaCl solution after removing the GFRP layer	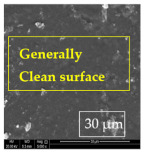	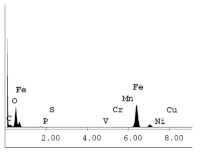
GFRP/steel pipes immersed for one year in H_2_SO_4_ solution after removing the GFRP layer	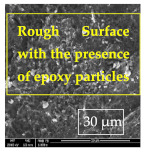	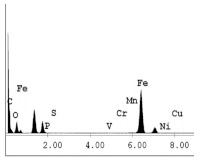
GFRP/steel pipes immersed for one year in HCl solution after removing the GFRP layer	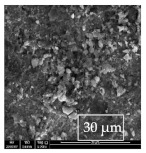	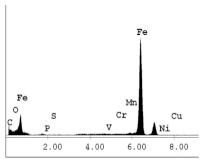
Steel pipes immersed for two weeks in NaCl solution	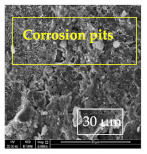	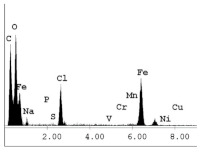
Steel pipes immersed for two weeks in H_2_SO_4_ solution	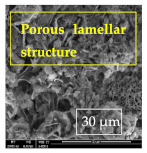	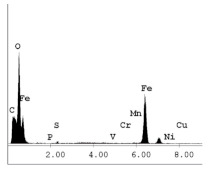
Steel pipes immersed for two weeks in HCl solution	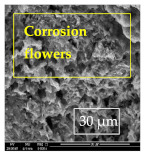	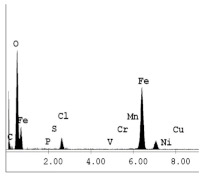

## Data Availability

The data presented in this study are available on request from the corresponding author.
